# Inverse relationship between *Fusobacterium nucleatum* amount and tumor CD274 (PD‐L1) expression in colorectal carcinoma

**DOI:** 10.1002/cti2.1453

**Published:** 2023-08-02

**Authors:** Tomotaka Ugai, Takashi Shimizu, Hidetaka Kawamura, Satoko Ugai, Yasutoshi Takashima, Genki Usui, Juha P Väyrynen, Kazuo Okadome, Koichiro Haruki, Naohiko Akimoto, Yohei Masugi, Annacarolina da Silva, Kosuke Mima, Xuehong Zhang, Andrew T Chan, Molin Wang, Wendy S Garrett, Gordon J Freeman, Jeffrey A Meyerhardt, Jonathan A Nowak, Mingyang Song, Marios Giannakis, Shuji Ogino

**Affiliations:** ^1^ Program in MPE Molecular Pathological Epidemiology, Department of Pathology Brigham and Women's Hospital and Harvard Medical School Boston MA USA; ^2^ Department of Epidemiology Harvard T.H. Chan School of Public Health Boston MA USA; ^3^ Department of Medical Oncology Dana‐Farber Cancer Institute and Harvard Medical School Boston MA USA; ^4^ Cancer and Translational Medicine Research Unit, Medical Research Center Oulu Oulu University Hospital and University of Oulu Oulu Finland; ^5^ Department of Pathology and Laboratory Medicine Weill Cornell Medicine New York NY USA; ^6^ Department of Gastroenterological Surgery, Graduate School of Medical Sciences Kumamoto University Kumamoto Japan; ^7^ Channing Division of Network Medicine, Department of Medicine Brigham and Women's Hospital and Harvard Medical School Boston MA USA; ^8^ Department of Nutrition Harvard T.H. Chan School of Public Health Boston MA USA; ^9^ Clinical and Translational Epidemiology Unit Massachusetts General Hospital and Harvard Medical School Boston MA USA; ^10^ Division of Gastroenterology Massachusetts General Hospital Boston MA USA; ^11^ Department of Immunology and Infectious Diseases Harvard T.H. Chan School of Public Health Boston MA USA; ^12^ Department of Biostatistics Harvard T.H. Chan School of Public Health Boston MA USA; ^13^ Department of Molecular Metabolism Harvard T.H. Chan School of Public Health Boston MA USA; ^14^ Harvard T.H. Chan Microbiome in Public Health Center Boston MA USA; ^15^ Broad Institute of MIT and Harvard Cambridge MA USA; ^16^ Department of Medicine Brigham and Women's Hospital and Harvard Medical School Boston MA USA; ^17^ Cancer Immunology and Cancer Epidemiology Programs Dana‐Farber Harvard Cancer Center Boston MA USA

**Keywords:** colorectal neoplasm, immune tolerance, immunology, microbiology, molecular pathological epidemiology

## Abstract

**Objectives:**

The CD274 (programmed cell death 1 ligand 1, PD‐L1)/PDCD1 (programmed cell death 1, PD‐1) immune checkpoint axis is known to regulate the antitumor immune response. Evidence also supports an immunosuppressive effect of *Fusobacterium nucleatum*. We hypothesised that tumor CD274 overexpression might be inversely associated with abundance of *F. nucleatum* in colorectal carcinoma.

**Methods:**

We assessed tumor CD274 expression by immunohistochemistry and *F. nucleatum* DNA within tumor tissue by quantitative PCR in 812 cases among 4465 incident rectal and colon cancer cases that had occurred in two prospective cohort studies. Multivariable logistic regression analyses with inverse probability weighting were used to adjust for selection bias because of tissue data availability and potential confounders including microsatellite instability status, CpG island methylator phenotype, LINE‐1 methylation level and *KRAS*, *BRAF* and *PIK3CA* mutations.

**Results:**

*Fusobacterium nucleatum* DNA was detected in tumor tissue in 109 (13%) cases. Tumor CD274 expression level was inversely associated with the amount of *F. nucleatum* in colorectal cancer tissue (*P* = 0.0077). For one category‐unit increase in three ordinal *F. nucleatum* categories (negative vs. low vs. high), multivariable‐adjusted odds ratios (with 95% confidence interval) of the low, intermediate and high CD274 categories (vs. negative) were 0.78 (0.41–1.51), 0.64 (0.32–1.28) and 0.50 (0.25–0.99), respectively (*P*_trend_ = 0.032).

**Conclusions:**

Tumor CD274 expression level was inversely associated with the amount of *F. nucleatum* in colorectal cancer tissue, suggesting that different immunosuppressive mechanisms (i.e. PDCD1 immune checkpoint activation and tumor *F. nucleatum* enrichment) tend to be used by different tumor subgroups.

## Graphical abstract

In this study, we found that tumor CD274 expression level was inversely associated with the amount of *Fusobacterium nucleatum* in colorectal cancer tissue. Our finding suggests that different immunosuppressive mechanisms (i.e. PDCD1 immune checkpoint activation and tumor *F. nucleatum* enrichment) tend to be used by different tumor subgroups.

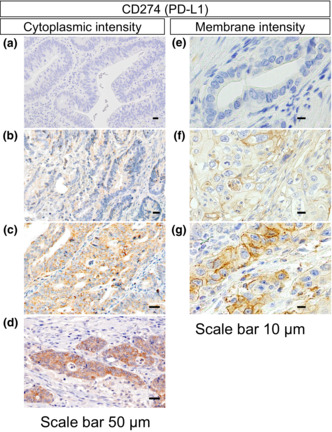

## Introduction

Immune checkpoint represents body's natural mechanism of regulating T‐cell‐mediated immune response.^1^ This mechanism functions in certain tumors to evade from antitumor immune response.^1^ In particular, the CD274 (HGNC:17635; programmed cell death 1 ligand 1, PD‐L1)/PDCD1 (HGNC:8760; programmed cell death 1, PD‐1) axis is one of the most intensively studied immune checkpoints.^2^, ^3^, ^4^, ^5^, ^6^, ^7^ A subset of colorectal carcinomas have been shown to overexpress CD274 (PD‐L1), which may contribute to tumor immunoevasion.^2^, ^3^, ^4^, ^5^, ^6^, ^7^ Tumor CD274 overexpression has been inversely associated with FOXP3^+^ regulatory T‐cell (Treg) density in colorectal carcinoma, suggesting that tumor CD274 expression and Treg infiltrates might be mutually exclusive immunoevasion mechanisms.^2^

Accumulating evidence indicates the pathogenic interactions between neoplastic cells, immune cells, microbiota and other components in the tumor microenvironment in colorectal carcinoma.^8^, ^9^ Among various micro‐organisms, *Fusobacterium nucleatum* is considered to contribute to colorectal carcinogenesis.^10^, ^11^, ^12^ Studies have shown that *F. nucleatum* inhibits T‐cell‐mediated antitumor immune responses,^12^, ^13^, ^14^, ^15^, ^16^ suggesting *F. nucleatum* abundance as one of immune evasion mechanisms in colorectal cancer. One study demonstrated that tumor abundance of *F. nucleatum* was associated inversely with FOXP3^+^ cell infiltrates in colorectal cancer.^16^

Based on these findings, we hypothesised that tumor CD274 overexpression might be inversely associated with abundance of *F. nucleatum* in colorectal cancer. We tested our hypothesis utilising data from more than 4400 colon and rectal adenocarcinomas, including 812 tumors with available data on CD274 expression and tissue *F. nucleatum*, within two prospective cohort studies. Our integrative data set allowed us to examine the relationship between tumor CD274 expression and the tissue abundance of *F. nucleatum* while adjusting for selection bias and potential confounders.

## Results

During the follow‐up period of the two USA‐wide prospective cohort studies, we identified 4465 incident colorectal adenocarcinoma cases, including 812 cases with available data on tumor CD274 (PD‐L1) expression and *F. nucleatum* in tumor tissue. Table 1 shows clinical, pathological and molecular characteristics of the 812 cases. Using the two‐sided α level of 0.005, *F. nucleatum* abundance was significantly associated with poor tumor differentiation, CpG island methylator phenotype (CIMP)‐high status and microsatellite instability (MSI)‐high status, whereas tumor CD274 overexpression was associated with non‐MSI‐high status. Tumor CD274 expression level was inversely associated with the amount of *F. nucleatum* in colorectal cancer tissue with nonsignificant but suggestive evidence at the stringent α level of 0.005 (*P* = 0.0077).

**Table 1:** Clinical, pathological and molecular characteristics of colorectal cancer cases according to the amount of tumor *Fusobacterium nucleatum* DNA and tumor CD274 (HGNC:17635; PD‐L1) expression level

Characteristic^a^	*F. nucleatum*					Tumor CD274 expression score				
	All cases (*N* = 812)	Negative (*N* = 703)	Low (*N* = 55)	High (*N* = 54)	*P‐*value^b^	Negative (score 0; *N* = 93)	Low (score 1; *N* = 231)	Intermediate (score 2; *N* = 210)	High (score 3–5; *N* = 278)	*P‐*value^b^
Sex										
Male (HPFS)	361 (44%)	311 (44%)	28 (51%)	22 (41%)	0.34	46 (49%)	104 (45%)	101 (48%)	110 (40%)	0.19
Female (NHS)	451 (56%)	392 (56%)	22 (49%)	32 (59%)		47 (51%)	127 (55%)	109 (52%)	168 (60%)	
Age (years) (Median, IQR)	70 (64–76)	69 (63–76)	70 (65–77)	71 (64–76)	0.32	71 (64–77)	69 (53–75)	71 (65–78)	68 (62–74)	0.12
Family history of colorectal cancer in a first‐degree relative										
Absent	639 (79%)	550 (79%)	45 (83%)	44 (83%)	0.60	75 (82%)	181 (79%)	169 (80%)	214 (78%)	0.90
Present	165 (21%)	147 (21%)	9 (17%)	9 (17%)		17 (18%)	48 (21%)	41 (20%)	59 (22%)	
Tumor location										
Proximal colon	412 (51%)	346 (50%)	34 (62%)	32 (59%)	0.13	43 (46%)	128 (56%)	103 (50%)	138 (50%)	0.76
Distal colon	236 (29%)	216 (31%)	7 (13%)	13 (24%)		22 (24%)	57 (25%)	66 (32%)	91 (33%)	
Rectum	160 (20%)	137 (20%)	14 (25%)	9 (17%)		28 (30%)	44 (19%)	39 (19%)	49 (18%)	
Tumor differentiation										
Well to moderate	735 (91%)	649 (92%)	45 (83%)	41 (76%)	< 0.0001	79 (85%)	208 (90%)	194 (92%)	254 (91%)	0.21
Poor	76 (9%)	54 (8%)	9 (17%)	13 (24%)		14 (15%)	22 (10%)	16 (8%)	24 (9%)	
AJCC disease stage										
I	172 (23%)	159 (24%)	7 (14%)	6 (12%)	0.20	23 (28%)	52 (25%)	46 (23%)	51 (20%)	0.0088
II	246 (33%)	204 (31%)	19 (37%)	23 (44%)		27 (33%)	74 (35%)	69 (35%)	76 (29%)	
III	222 (29%)	188 (29%)	21 (41%)	13 (25%)		24 (30%)	53 (25%)	55 (28%)	90 (34%)	
IV	113 (15%)	99 (15%)	4 (8%)	10 (19%)		7 (9%)	33 (16%)	29 (15%)	44 (17%)	
CIMP status										
Negative/Low	584 (78%)	520 (81%)	36 (73%)	28 (56%)	0.0002	59 (72%)	165 (78%)	144 (77%)	216 (82%)	0.21
High	160 (22%)	125 (19%)	13 (27%)	22 (44%)		23 (28%)	46 (22%)	44 (23%)	47 (18%)	
MSI status										
Non‐MSI‐high	649 (82%)	580 (85%)	36 (68%)	33 (61%)	< 0.0001	63 (69%)	184 (82%)	164 (82%)	238 (87%)	0.0016
MSI‐high	138 (18%)	100 (15%)	17 (32%)	21 (39%)		28 (31%)	40 (18%)	35 (18%)	35 (13%)	
LINE‐1 methylation level (median, IQR)	63 (57–69)	62 (57–69)	62 (55–69)	66 (61–70)	0.053	65 (60–71)	62 (56–69)	64 (57–70)	62 (56–68)	0.035
*KRAS* mutation										
Wild‐type	465 (59%)	407 (60%)	24 (46%)	34 (65%)	0.10	63 (69%)	133 (59%)	114 (57%)	155 (58%)	0.21
Mutant	319 (41%)	273 (40%)	28 (54%)	18 (35%)		28 (31%)	91 (41%)	86 (43%)	114 (42%)	
*BRAF* mutation										
Wild‐type	670 (85%)	587 (86%)	45 (85%)	38 (70%)	0.010	76 (83%)	186 (83%)	173 (87%)	235 (86%)	0.62
Mutant	121 (15%)	97 (14%)	8 (15%)	16 (30%)		16 (17%)	39 (17%)	27 (14%)	39 (14%)	
*PIK3CA* mutation										
Wild‐type	633 (85%)	549 (86%)	40 (83%)	44 (85%)	0.91	74 (86%)	191 (88%)	162 (85%)	206 (83%)	0.45
Mutant	109 (15%)	93 (14%)	8 (17%)	8 (15%)		12 (14%)	26 (12%)	28 (15%)	43 (17%)	
Tumor CD274 (PD‐L1) expression level										
Negative (score 0)	93 (11%)	74 (11%)	9 (16%)	10 (19%)	0.0077					
Low (score 1)	231 (28%)	195 (28%)	17 (31%)	19 (35%)						
Intermediate (score 2)	210 (26%)	185 (26%)	10 (18%)	15 (28%)						
High (score 3–5)	278 (34%)	249 (35%)	19 (35%)	10 (19%)						

AJCC, American Joint Committee on Cancer; CIMP, CpG island methylator phenotype; HPFS, Health Professionals Follow‐up Study; IQR, interquartile range; LINE‐1, long interspersed nucleotide element‐1; MSI, microsatellite instability; NHS, Nurses' Health Study; SD, standard deviation.

^a^ Percentage indicates the proportion of cases with a specific clinical, pathologic, or molecular characteristic among all colorectal cancer cases or in strata of *F. nucleatum* DNA level and tumor CD274 expression level.

^b^ The chi‐square test or Fisher's exact test was applied to compare categorical data. The Spearman correlation test was also used to compare continuous (age and LINE‐1) or ordinal categorical variables (tumor locations and disease stage).

In our primary hypothesis testing, we conducted an ordinal logistic regression analysis to assess the association of tumor CD274 expression with the amount of tumor *F. nucleatum* DNA (Table 2). Tumor CD274 expression level was inversely associated with the amount of *F. nucleatum* in colorectal cancer tissue. Multivariable‐adjusted odds ratios (95% confidence interval) of the low, intermediate and high CD274 expression categories (vs. negative) for one category‐unit increase in three ordinal *F. nucleatum* categories (negative, low and high) were 0.78 (0.41–1.51), 0.64 (0.32–1.28) and 0.50 (0.25–0.99), respectively, with nonsignificant but suggestive evidence (*P*_trend_ = 0.032).

**Table 2:** Inverse probability weighting‐adjusted ordinal logistic regression analysis to assess the association of tumor CD274 (PD‐L1) expression level (predictor) with tumor *Fusobacterium nucleatum* (outcome)

	*F. nucleatum* DNA amount	
	For one category‐unit increase in three ordinal categories	
	Univariable odds ratio (95% CI)^a^	Multivariable odds ratio (95% CI)^a^ ^,^ ^b^
CD274 (PD‐L1) expression level		
Negative (*N* = 93)	1 (reference)	1 (reference)
Low (*N* = 231)	0.67 (0.35–1.27)	0.78 (0.41–1.51)
Intermediate (*N* = 210)	0.53 (0.27–1.05)	0.64 (0.32–1.28)
High (*N* = 278)	0.40 (0.21–0.79)	0.50 (0.25–0.99)
*P*_trend_ ^c^	0.0069	0.032
MSI status		
Non‐MSI‐high	1 (reference)	1 (reference)
MSI‐high	3.47 (2.18–5.52)	3.23 (2.03–5.15)

CI, confidence interval; MSI, microsatellite instability.

^a^ Inverse probability weighting method was applied to reduce selection bias because of the availability of data on CD274 expression and *F. nucleatum*.

^b^ Multivariable ordinal logistic regression model initially included sex, age, year of diagnosis, family history of colorectal cancer in a first‐degree relative(s), tumor location, MSI status, CpG island methylator phenotype status, long‐interspersed nucleotide element‐1 methylation level, *KRAS*, *PIK3CA*, and *BRAF* mutations. A backward elimination with a threshold *P* of 0.05 was used to select variables for the final model. The MSI status was the only covariate that remained in the final model.

^c^ *P*_trend_ value was calculated by the linear trend across the ordinal categories of the tumor CD274 expression level (as an ordinal‐scale predictor variable) in the ordinal logistic regression model.

We confirmed that similar results were observed by sensitivity analyses without inverse probability weighting adjustment (Supplementary table 1). Moreover, we conducted a logistic regression analysis to assess the association using binary categorisation for *F. nucleatum* (positive vs. negative) and observed similar findings to our main findings by an ordinal logistic regression analysis (Supplementary table 2).

As secondary analyses, we examined statistical interaction between tumor CD274 expression and MSI status for tumor *F. nucleatum* status. A statistically significant interaction was not observed between tumor CD274 expression and MSI status (Table 3).

**Table 3:** Association of tumor CD274 (PD‐L1) expression level (predictor) with tumor *Fusobacterium nucleatum* (outcome) in relation to MSI status

	*F. nucleatum* DNA amount
	For one category‐unit increase in three ordinal categories
	Multivariable odds ratio (95% CI)^a^ ^,^ ^b^
Non‐MSI‐high	
CD274 (PD‐L1) expression level	
Negative (*N* = 63)	1 (reference)
Low (*N* = 184)	0.64 (0.28–1.47)
Middle (*N* = 164)	0.60 (0.25–1.41)
High (*N* = 238)	0.43 (0.18–1.01)
*P*_trend_ ^c^	0.065
MSI‐high	
CD274 (PD‐L1) expression level	
Negative (*N* = 30)	1 (reference)
Low (*N* = 47)	1.13 (0.39–3.30)
Middle (*N* = 46)	0.62 (0.18–2.17)
High (*N* = 40)	0.53 (0.17–1.68)
*P*_trend_ ^c^	0.15
*P*_interaction_ ^d^	0.86

MSI, microsatellite instability.

^a^ Inverse probability weighting was applied to reduce selection bias because of the availability of data on CD274 expression and *F. nucleatum*.

^b^ Multivariable ordinal logistic regression model initially included sex, age, year of diagnosis, family history of colorectal cancer, tumor location, CpG island methylator phenotype, long‐interspersed nucleotide element‐1 methylation level, *KRAS*, *PIK3CA*, and *BRAF* mutations. A backward elimination with a threshold *P* of 0.05 was used to select variables for the final model. Any variable did not remain in the final model.

^c^ *P*_trend_ was calculated by the linear trend across the ordinal categories of the tumor CD274 expression level (as an ordinal‐scale predictor variable) in the ordinal logistic regression model.

^d^ *P*_interaction_ was calculated using the Wald test for the cross‐product term of the tumor CD274 expression level (as an ordinal‐scale predictor variable) and MSI status (MSI‐high vs. non‐MSI‐high as a binary variable) in the ordinal logistic regression model.

## Discussion

In this study, we found that tumor CD274 (PD‐L1) expression was inversely associated with tissue *F. nucleatum* abundance in colorectal carcinoma, independent of other clinical and molecular features including MSI status. To our knowledge, our analysis is the largest human population study to show the inverse relationship between tumor CD274 expression and tumor *F. nucleatum* amount.

Accumulating evidence indicates that *F. nucleatum* suppresses the adaptive T‐cell response.^17^ Supporting this, our previous study showed an inverse association of tissue *F. nucleatum* abundance with tumor stromal densities of CD3^+^ cell and CD3^+^CD4^+^CD45RO^+^ cells.^15^ *F. nucleatum* abundance has been associated with MSI‐high status in colorectal cancer.^18^, ^19^ Notably, *F. nucleatum* seems to have differential effects on immune cell infiltrates by tumor MSI status; it has been associated with lower immune infiltrates in MSI‐high tumors, but with higher immune infiltrates in non‐MSI‐high tumors.^20^ The tumor MSI status is one of major biomarkers in colorectal carcinoma and is associated with a number of clinical and pathological features.^21^, ^22^ In average, MSI‐high tumors tend to show much higher immune reaction than non‐MSI‐high tumors. It has been speculated that *F. nucleatum* may function as an immunosuppressor in MSI‐high tumors but as a proinflammatory agent in non‐MSI‐high tumors.^20^ Our study also showed that tumor CD274 overexpression was significantly associated with non‐MSI‐high colorectal cancer. Therefore, the immunosuppressive role of tumor CD274 overexpression is likely more prominent in non‐MSI‐high tumors, whereas that of *F. nucleatum* is more prominent in MSI‐high tumors.

Recent studies reported that *F. nucleatum* induced CD274 (PD‐L1) expression in colorectal cancer cells^23^, ^24^ and other cancer cells (including head and neck cancer cells and oral cancer cells),^25^, ^26^ which is not consistent with our findings of the anticorrelation between *F. nucleatum* abundance and tumor CD274 expression. Results from those studies using experimental models need to be interpreted cautiously, because even the best experimental model cannot exactly replicate complex human tumor microenvironment. Furthermore, although one study^24^ used human colorectal cancer tissue specimens, its sample size was quite small (*n* = 34), which could suffer from selection bias and might not yield robust reproducible findings. In the study^24^ conducted by Gao *et al*., only membrane CD274 expression was evaluated. By contrast, we evaluated both cytoplasm and membrane CD274 expression levels and used a large number of human tissue specimens. We also adjusted for potential confounders and selection bias because of tissue data availability using the inverse probability weighting method. The conflicting results are possibly because of differences in organ sites, methods, study designs and study populations. Although we rigorously investigated the association with adjustment for potential confounders and selection bias, our findings should be validated in independent data sets.

Evidence indicates that the PI3K/AKT signalling pathway activation via somatic *PIK3CA* mutations and loss of PTEN expression has been linked to tumor CD274 upregulation in experimental studies^27^, ^28^, ^29^, ^30^ and a human colorectal tissue analysis.^31^ By contrast, *F. nucleatum* likely exert carcinogenic effects through activation of the CTNNB1/WNT signalling pathway, which can result in suppression of antitumor immunity.^13^, ^17^, ^32^, ^33^, ^34^ These differential mechanistic links may underlie the observed inverse association of tumor CD274 upregulation and *F. nucleatum* abundance.

Colorectal cancer enriched with *F. nucleatum* represents a tumor subtype associated with proximal location (particularly cecum), poor differentiation, high stage and worse survival.^18^, ^19^, ^35^ A previous molecular pathological epidemiology (MPE) study suggests that this colorectal cancer subtype might be caused by diets that correlate with systemic inflammatory status.^36^ Additional MPE research^37^ and experimental studies are needed to elucidate how diets modify cancer risks. Moreover, it is desirable to identify a preventative measure specific for this aggressive cancer subtype beyond regular cancer screening programmes. A study demonstrated that the so‐called prudent diet, which is rich in whole grains, vegetables and legumes, was associated with lower incidence of colorectal cancer enriched with *F. nucleatum* but not with incidence of colorectal cancer without detectable *F. nucleatum*.^38^ Hence, a dietary modification (prudent and anti‐inflammatory diets) may be an attractive preventative strategy for *F. nucleatum*‐rich colorectal cancer.

Although tumor CD274 (PD‐L1) overexpression does not appear to have a prognostic role in colorectal carcinoma,^2^, ^6^ our previous study indicates that tumor CD274 overexpression may serve as a predictive biomarker for tumor resistance to aspirin use after colorectal cancer diagnosis.^39^ Aspirin has shown potential efficacy in prolonging survival of a subset of colorectcal cancer patients, especially those with tumor *PIK3CA* mutation.^40^, ^41^, ^42^ A predictive role of tumor CD274 overexpression in specific cancer patients needs further investigations.

Limitations in this study should be acknowledged. Laboratory procedures including formalin fixation, paraffin embedding and storage might likely have affected the quantification and detection rates of quantitative PCR despite all efforts to minimise such effects. Nonetheless, in our validation study using this quantitative PCR assay, we showed a reasonably high concordance of the detection of *F. nucleatum* in paired frozen tissue and FFPE specimens.^16^ Our method also showed high reproducibility (interassay coefficient of variation ≤ 1%) in measuring the amount of *F. nucleatum* in FFPE tissue specimens.^16^ Furthermore, to validate our data on *F. nucleatum*, we amplified and sequenced the V3 region of bacterial 16S rRNA gene. A positive/negative concordance rate between 16S rRNA sequencing data and quantitative PCR data was high (83.8%).

The present study has notable strengths. First, we integrated clinical, pathological and tumor molecular data and then rigorously investigated the relationship between CD274 expression level and *F. nucleatum* in colorectal cancer tissue while adjusting for potential confounders, including MSI status. Consequently, this is the largest and first study to assess such a relationship with a sufficient sample size. Second, we adjusted for selection bias (because of tissue data availability) utilising the 4465 incident colorectal cancer cases that occurred in our prospective cohort studies. As sensitivity analyses, we also confirmed that similar results were observed without inverse probability weighting adjustment, suggesting no substantial selection bias. These analyses are particularly important because selection bias can occur and significantly affect findings in observational studies.^43^ Third, we utilised data of colorectal cancer cases that occurred in large‐scale USA‐wide prospective cohort studies. Therefore, our cases came from many hospitals throughout the United States, which increases the generalisability of our findings.

In conclusion, we found that tumor CD274 expression level was inversely associated with the amount of *F. nucleatum* in colorectal cancer tissue. Our findings suggest that different immunosuppressive mechanisms (i.e. PDCD1 immune checkpoint activation and tumor *F. nucleatum* enrichment) tend to be used by different tumor subgroups.

## Methods

### Use of standardised official symbols

We use HUGO (Human Genome Organisation)‐approved official symbols (or root symbols) for genes and gene products, including AKT, BRAF, CACNA1G, CD274, CDKN2A, CRABP1, CTNNB1, IGF2, KRAS, MLH1, NEUROG1, PDCD1, PIK3CA, PTEN, RUNX3, SLCO2A1, SOCS1 and WNT, all of which are described at www.genenames.org. Gene symbols are italicised, whereas symbols for gene products are not italicised.

### Study population

**Figure 1:**
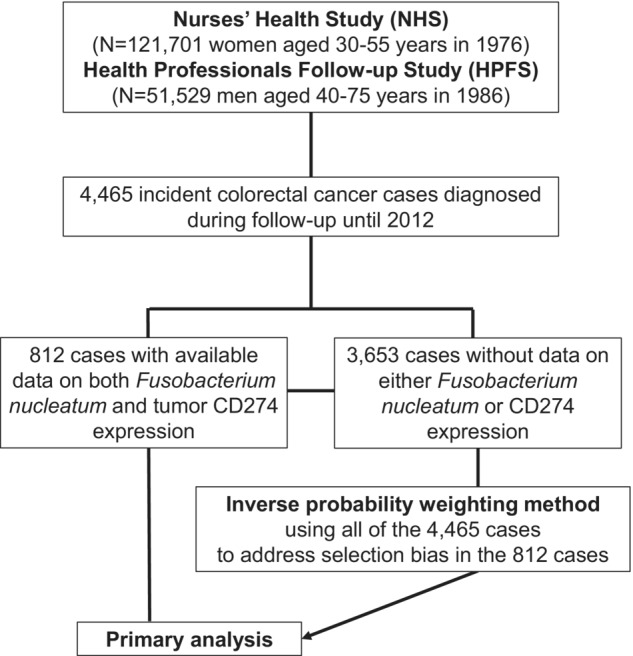
A flow diagram of the study population in the Nurses' Health Study and Health Professionals Follow‐up Study.

We utilised data from the Health Professionals Follow‐up Study, which had followed 51 529 male physicians and other health professionals aged 40–75 years in 1986 and Nurses' Health Study (which had followed 121 701 female nurses aged 30–55 years in 1976).^44^ Every 2 years, we sent follow‐up questionnaires to update information, such as lifestyles and newly diagnosed diseases, including cardiovascular disease and cancer. We had a high response rate (> 90%) for these questionnaires in each follow‐up questionnaire cycle. The National Death Index was applied to confirm the deaths of participants and identify unreported colorectal cancer cases. Study staff physicians reviewed medical records, confirmed a diagnosis of colorectal adenocarcinoma in each patient and recorded data on several pertinent variables, including primary tumor location, tumor size, pT stage, pN stage, M stage (and causes of mortality for lethal cancer cases). In the Nurses' Health Study and Health Professionals Follow‐up Study, we identified 4465 colorectal cancer cases during the follow‐up until 2012. In this study, we utilised 812 colorectal cancer cases with available data on CD274 expression and the amount of *F. nucleatum* (Table 1). We further utilised clinical data of the 3653 cases without available CD274 or *Fusobacterium* data to adjust for selection bias caused by tissue data availability (Figure 1). The colorectal continuum theory allowed for the inclusion of both colon and rectal carcinomas in this analysis.^45^

Informed consent was obtained from patients (or next of kin for deceased patients) to use tissue specimens for molecular pathological analyses. The study protocol was approved by the institutional review boards of Harvard T.H. Chan School of Public Health and Brigham and Women's Hospital (Boston, MA, USA), and those of participating registries (see the Acknowledgments section for the details) as required.

### Tumor tissue analyses

We obtained formalin‐fixed paraffin‐embedded tumor tissue samples from hospitals where surgical tumor resections were performed on the study participants with colorectal cancer. The study pathologist (SO), who was blinded to other data, reviewed histopathological sections from collected colorectal cancer cases and recorded pathological features.^46^

Tumor and normal DNA specimens were extracted from FFPE tissue sections using QIAamp DNA FFPE Tissue Kit and GeneRead DNA FFPE Kit (Qiagen, Hilden, Germany). Microsatellite instability status was determined using polymerase chain reaction (PCR) on microsatellite markers (D2S123, D5S346, D17S250, BAT25, BAT26, BAT40, D18S55, D18S56, D18S67 and D18S487); instability in ≥ 30% of the markers indicated MSI‐high status.^46^ We measured methylation levels of eight promoter CpG islands specific for CIMP (CACNA1G, CDKN2A, CRABP1, IGF2, MLH1, NEUROG1, RUNX3 and SOCS1) and long interspersed nucleotide element‐1 as previously described.^47^, ^48^ The presence of ≥ 5/8 hypermethylated CpG islands indicated CIMP‐high status. PCR followed by pyrosequencing was conducted to identify mutations in *BRAF* (codon 600), *KRAS* (codons 12, 13, 61 and 146) and *PIK3CA* (exons 9 and 20).^42^, ^49^

To measure the relative amount of *F. nucleatum* DNA in tumor tissue, we performed quantitative PCR that targeted the *nusG* gene using *SLCO2A1* as the human reference gene.^16^, ^50^ Using the StepOnePlus real‐time PCR Systems (Applied Biosystems), the normalised amount of *F. nucleatum* DNA was calculated as $2^{-\Delta C_{t}}$, where ∆*C*_t_ is the mean *C*_t_ value of *F. nucleatum* minus the mean *C*_t_ value of *SLCO2A1*. Cases with detectable *F. nucleatum* DNA were dichotomised (low vs. high) based on the median cut‐off, while cases without detectable *F. nucleatum* were categorised as negative.

**Figure 2:**
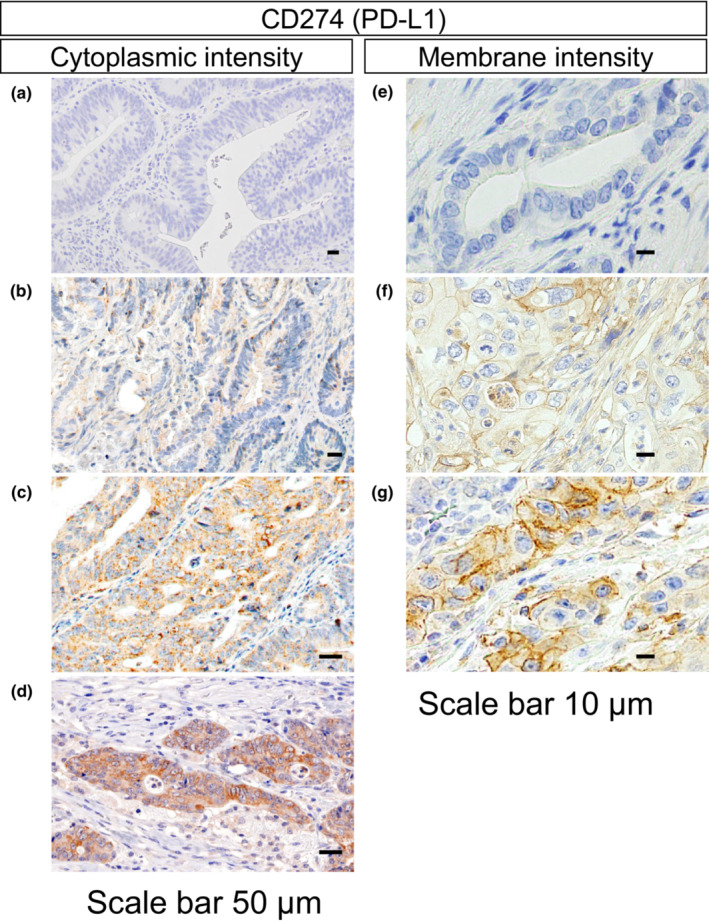
Tumor CD274 (HGNC:17635; PD‐L1) expression in colorectal carcinoma. Tumor CD274 (PD‐L1) expression was evaluated based on immunohistochemical staining in the cytoplasm and membrane of tumor cells. Cytoplasmic expression level (as an overall pattern) was scored as 0 (negative, **a**), 1 (low, **b**), 2 (intermediate, **c**), or 3 (high, **d**). Membrane CD274 expression level (as an overall pattern) in tumor cells was scored as 0 (negative, **e**), 1 (low, **f**) or 2 (high, **g**). The black bar indicates 50 μm in **a–d** and 10 μm in **e–g**.

Study staff constructed tissue microarrays using collected formalin‐fixed paraffin‐embedded tissue.^46^ To evaluate CD274 (HGNC:17635; programmed cell death 1 ligand 1, PD‐L1) expression, immunohistochemical analyses were performed using an anti‐CD274 (PD‐L1) antibody (Clone MIH1, dilution, 1:50; eBioscience, San Diego, CA, USA), as previously described.^2^ Overall, representative cytoplasmic expression level (intensity) was scored as 0 (negative), 1 (low), 2 (intermediate) or 3 (high), while membrane expression level was scored as 0 (negative), 1 (low) or 2 (high) in a given tumor (Figure 2). Tumor CD274 expression score (ranging from 0 to 5) was the sum of the cytoplasmic and membrane expression scores.^2^ Considering the number of cases in each score category, we used tumor CD274 expression status in each tumor as negative (score 0), low (score 1), intermediate (score 2) or high (score 3–5). Appropriate positive and negative controls existed and worked as expected in each run of immunohistochemistry. One of the study pathologists (YM) interpreted CD274 expression level in all cases. For an agreement study, a second pathologist (AdS) independently reviewed 148 cases for CD274 expression, and the weighted kappa value between the two independent pathologists was 0.65 (*P* < 0.001).

### Statistical analysis

We used the SAS software (version 9.4; SAS Institute, Cary, NC, USA) for all statistical analyses. We used the two‐sided α level of 0.005 for our primary hypothesis testing, as recommended by expert statisticians.^51^ Our primary hypothesis was that tumor CD274 overexpression might be inversely associated with tumor *F. nucleatum* abundance. All other analyses were secondary analyses. The chi‐squared test or Fisher's exact test (if appropriate) was applied to compare categorical data. The Spearman correlation test was also used to compare continuous or ordinal categorical variables.

For our primary hypothesis testing, we constructed a multivariable ordinal logistic regression model that had tumor CD274 expression score (negative, low, intermediate, and high; an ordinal scale) as a predictor variable and *F. nucleatum* abundance (negative, low and high; an ordinal scale) as an outcome variable. *P* for trend was calculated by the linear trend across the ordinal categories of the tumor CD274 expression level (as an ordinal‐scale predictor variable) in the ordinal logistic regression model. As secondary analyses, we examined statistical interaction between tumor CD274 expression and MSI status for tumor *F. nucleatum* status using the Wald test for the cross‐product term. To adjust for selection bias (because of the availability of tumor tissue data), we integrated the inverse probability weighting method into the logistic regression models using demographic and clinical data of all 4465 colorectal cancer cases.^43^, ^52^ Multivariable inverse probability weighting‐adjusted logistic regression models initially included age at diagnosis (continuous), sex (female vs. male), year of diagnosis (continuous), family history of colorectal cancer in any first‐degree relative (present vs. absent), primary tumor location (proximal colon vs. distal colon vs. rectum), *BRAF* mutation status (mutant vs. wild‐type), *KRAS* mutation status (mutant vs. wild‐type), *PIK3CA* mutation status (mutant vs. wild‐type), MSI status (MSI‐high vs. non‐MSI‐high), CIMP status (high vs. low/negative) and long interspersed nucleotide element‐1methylation level (continuous). To select variables for the final model, a threshold of *P* = 0.05 was set in a backward stepwise elimination procedure. Cases with missing data (family history of colorectal cancer in any first‐degree relative, 1.0%; MSI status, 7.3%; CIMP status, 8.4%; *BRAF* mutation, 2.6%; *KRAS* mutation, 3.5%; and *PIK3CA* mutation, 8.6%) were included in the majority category of a given categorical covariate to limit degrees of freedom of the models. A separate indicator variable was used for cases with missing data on a continuous variable [long interspersed nucleotide element‐1 level (2.8%)] and an ordinal variable [tumor location (0.5%)]. We confirmed that our findings did not substantially change after excluding the cases with missing values in any of the covariates. The only covariate which remained in the final model was the MSI status and effect sizes of this covariate is shown in Table 2. We verified that the proportional odds assumption was satisfied in the final multivariable ordinal logistic regression model (*P* > 0.05).

## Author contributions

**Tomotaka Ugai:** Conceptualization; data curation; formal analysis; funding acquisition; investigation; methodology; project administration; supervision; writing – original draft; writing – review and editing. **Takashi Shimizu:** Formal analysis; methodology; writing – original draft. **Hidetaka Kawamura:** Investigation; writing – review and editing. **Satoko Ugai:** Investigation; writing – review and editing. **Yasutoshi Takashima:** Investigation; writing – review and editing. **Genki Usui:** Writing – review and editing. **Juha P Väyrynen:** Writing – review and editing. **Kazuo Okadome:** Writing – review and editing. **Koichiro Haruki:** Writing – review and editing. **Naohiko Akimoto:** Writing – review and editing. **Yohei Masugi:** Data curation; writing – review and editing. **Annacarolina da Silva:** Data curation; writing – review and editing. **Kosuke Mima:** Data curation; writing – review and editing. **Xuehong Zhang:** Writing – review and editing. **Andrew T Chan:** Writing – review and editing. **Molin Wang:** Writing – review and editing. **Wendy S Garrett:** Writing – review and editing. **Gordon J Freeman:** Writing – review and editing. **Jeffrey A Meyerhardt:** Writing – review and editing. **Jonathan A Nowak:** Investigation; supervision; writing – review and editing. **Mingyang Song:** Supervision; writing – review and editing. **Marios Giannakis:** Supervision; writing – review and editing. **Shuji Ogino:** Conceptualization; funding acquisition; resources; supervision; writing – original draft.

## Conflict of interest

ATC previously served as a consultant for Bayer Healthcare and Pfizer Inc. MG was on an advisory board for AstraZeneca and receives research funding from Bristol‐Myers Squibb. JAM has received institutional research funding from Boston Biomedical, has served as an advisor/consultant to Ignyta and COTA Healthcare, and served on a grant review panel for the National Comprehensive Cancer Network funded by Taiho Pharmaceutical. This study was not funded by any of these commercial entities.

## Supporting information


Supplementary tables 1 and 2

## Data Availability

Because of participant confidentiality and privacy concerns, data are available upon reasonable written request. According to standard controlled access procedure, applications to use Nurses' Health Study and Health Professionals Follow‐up Study resources will be reviewed by our External Collaborators Committee for scientific aims, evaluation of the fit of the data for the proposed methodology, and verification that the proposed use meets the guidelines of the Ethics and Governance Framework and the consent that was provided by the participants. Investigators wishing to use Nurses' Health Study and Health Professionals Follow‐up Study data are asked to submit a brief description of the proposed project [go to https://www.nurseshealthstudy.org/researchers (contact email: nhsaccess@channing.harvard.edu) and https://sites.sph.harvard.edu/hpfs/for‐collaborators/ for details].
