# Supplementary material for: Inverse relationship between Fusobacterium nucleatum amount and tumor CD274 (PD‐L1) expression in colorectal carcinoma
Source: Clin Transl Immunology. 2023 Aug 2;12(8):e1453. doi: 10.1002/cti2.1453 (PMC10394676; doi:10.1002/cti2.1453)
Supplement: Supplementary file 1 — Supplementary tables 1 and 2 [file CTI2-12-e1453-s001.docx]

**Supplementary table 1.** Ordinal logistic regression analysis to assess the association of tumor CD274 (PD-L1) expression level with tumor *F. nucleatum* (outcome) without inverse probability weighting.

|  | *F. nucleatum* DNA amount  For one category increase in three ordinal categories | | |
| --- | --- | --- | --- |
|  | Univariable odds ratio  (95% CI) |  | Multivariable odds ratio  (95% CI)† |
| CD274 (PD-L1) expression level |  |  |  |
| Negative (N=93) | 1 (reference) |  | 1 (reference) |
| Low (N=231) | 0.72 (0.39-1.34) |  | 0.84 (0.44-1.59) |
| Middle (N=210) | 0.53 (0.28-1.03) |  | 0.63 (0.32-1.24) |
| High (N=278) | 0.45 (0.24-0.84) |  | 0.54 (0.28-1.05) |
| P_trend_‡ | 0.0078 |  | 0.034 |
|  |  |  |  |
| MSI status |  |  |  |
| Non-MSI-high | 1 (reference) |  | 1 (reference) |
| MSI-high | 3.26 (2.09-5.08) |  | 3.08 (1.95-4.86) |

† Multivariable ordinal logistic regression model initially included sex, age, year of diagnosis, family history of colorectal cancer in a first-degree relative(s), tumor location, microsatellite instability (MSI) status, CpG island methylator phenotype status, long-interspersed nucleotide element-1 methylation level, *KRAS*, *PIK3CA*, and *BRAF* mutations. A backward elimination with a threshold *P* of 0.05 was used to select variables for the final model. The MSI status was the only covariate that remained in the final model.

‡ P_trend_ value was calculated by the linear trend across the ordinal categories of the tumor CD274 expression level (as an ordinal-scale predictor variable) in the ordinal logistic regression model for the amount of *F. nucleatum* (0 to 2, as an ordinal outcome variable).

CI, confidence interval; MSI, microsatellite instability.

**Supplementary table 2.** Inverse probability weighting-adjusted logistic regression analysis to assess the association of tumor CD274 (PD-L1) expression level (predictor) with tumor *F. nucleatum* (outcome).

|  | *F. nucleatum* DNA (positive vs. negative) | | |
| --- | --- | --- | --- |
|  | Univariable odds ratio  (95% CI)† |  | Multivariable odds ratio  (95% CI)†‡ |
| CD274 (PD-L1) expression level |  |  |  |
| Negative (N=93) | 1 (reference) |  | 1 (reference) |
| Low (N=231) | 0.65 (0.34-1.26) |  | 0.77 (0.39-1.51) |
| Middle (N=210) | 0.52 (0.26-1.03) |  | 0.61 (0.30-1.25) |
| High (N=278) | 0.40 (0.20-0.79) |  | 0.50 (0.25-1.01) |
| P_trend_§ | 0.007 |  | 0.035 |
|  |  |  |  |
| MSI status |  |  |  |
| Non-MSI-high | 1 (reference) |  | 1 (reference) |
| MSI-high | 3.49 (2.18-5.56) |  | 3.24 (2.02-5.19) |

† Inverse probability weighting was applied to reduce selection bias due to the availability of data on CD274 expression and *F. nucleatum*.

‡ Multivariable logistic regression model initially included sex, age, year of diagnosis, family history of colorectal cancer in a first-degree relative(s), tumor location, microsatellite instability (MSI) status, CpG island methylator phenotype status, long-interspersed nucleotide element-1 methylation level, *KRAS*, *PIK3CA*, and *BRAF* mutations. A backward elimination with a threshold *P* of 0.05 was used to select variables for the final model. The MSI status was the only covariate that remained in the final model.

§ P_trend_ was calculated by the linear trend across the ordinal categories of the tumor CD274 expression level (as an ordinal-scale predictor variable) in the logistic regression model.

CI, confidence interval; MSI, microsatellite instability.
